# Dysregulated microRNA Expression in Serum of Non-Vaccinated Children with Varicella

**DOI:** 10.3390/v6041823

**Published:** 2014-04-22

**Authors:** Yuhua Qi, Zheng Zhu, Zhiyang Shi, Yiyue Ge, Kangchen Zhao, Minghao Zhou, Lunbiao Cui

**Affiliations:** Key Laboratory of Enteric Pathogenic Microbiology, Ministry of Health, Institute of Pathogenic Microbiology, Jiangsu Provincial Center for Disease Control and Prevention, 172 Jiangsu Rd, Nanjing 210009, China; E-Mails: qiyuhua@jscdc.cn (Y.Q.); zhengzhu@jscdc.cn (Z.Z.); shizhiyang@jscdc.cn (Z.S.); geyiyue@jscdc.cn (Y.G.); zhaokangchen@jscdc.cn (K.Z.)

**Keywords:** varicella, microRNA, biomarker, low-density array, qRT-PCR

## Abstract

Circulating microRNAs (miRNAs) may play an important role in pathogen-host interactions and can serve as molecular markers for the detection of infectious diseases. To date, the relationship between circulating miRNAs and varicella-zoster virus (VZV) caused varicella has not been reported. Using TaqMan Low-Density Array (TLDA) analysis, expression levels of miRNAs in serum samples from 29 patients with varicella and 60 patients with *Bordetella pertussis* (BP), measles virus (MEV) and enterovirus (EV) were analyzed. The array results showed that 247 miRNAs were differentially expressed in sera of the varicella patients compared with healthy controls (215 up-regulated and 32 down-regulated). Through the following qRT-PCR confirmation and receiver operational characteristic (ROC) curve analysis, five miRNAs (miR-197, miR-629, miR-363, miR-132 and miR-122) were shown to distinguish varicella patients from healthy controls and other microbial infections with moderate sensitivity and specificity. A number of significantly enriched pathways regulated by these circulating miRNAs were predicted, and some of them were involved in inflammatory response, nervous system and respiratory system development. Our results, for the first time, revealed that a number of miRNAs were differentially expressed during VZV infection, and these five serum miRNAs have great potential to serve as biomarkers for the diagnosis of VZV infection in varicella patients.

## 1. Introduction

Varicella, also called chickenpox, is a highly contagious disease caused by varicella-zoster virus (VZV) which belongs to the family of *alphaherpesviruses*. Varicella is often known as a mild illness in childhood. However, serious complications such as secondary bacterial skin and soft tissue infections, pneumonia, dehydration, and encephalitis can occur in patients [1,2]. Although varicella vaccination has become routine for all children at 12–15 months of age in the United States, Germany, Australia, and Korea, outbreaks of varicella are still seen in the community [3,4]. Other countries, such as China and Netherland, have not incorporated varicella vaccination of children into the national childhood immunization schedules [3,5,6]. The incidence of varicella in China is increasing annually [7,8]. A recent study reported that the children under 1 year of age had higher risk for hospitalization compared with varicella-infected children aged 5–9 years [9]. Therefore, varicella remains an important public health problem worldwide, a rapid and reliable diagnostic method is essential for appropriate treatment and prophylaxis. The specific diagnostic tests available for varicella are mainly cell culture, direct fluorescent antibody (DFA) testing, serological tests and polymerase chain reaction (PCR). Culture is not used currently because of its lower sensitivity and longer incubation time (5–14 days) [10,11]. DFA and serological tests are time-consuming and require further confirmation [12]. PCR brings great progress in varicella rapid diagnosis [13,14,15]. However, laboratory contamination is of greater concern with PCR than other diagnostic methods.

MicroRNAs (miRNAs) are endogenous, small (18–25 nt), non-coding, and highly conserved RNAs that specifically regulate gene expression and play important roles in a variety of cellular functions [16,17]. Recently, circulating miRNAs as novel and non-invasive biomarkers for disease diagnosis have been intensively studied in many areas, such as various cancers, pregnancy, diabetes, psychosis, and various infectious diseases [18,19,20,21,22,23,24,25]. Serum miRNAs are present in stable forms which are protected from endogenous RNase activity [26,27]. Their expression levels were consistent among individuals of the same species [26]. Serum, plasma, and other fluid specimens are readily available and non-invasive. These unique characteristics make circulating miRNAs become useful biomarkers for disease diagnosis. However, serum/plasma miRNAs as novel biomarkers for the detection of infectious diseases, such as viral or common bacterial infections, remain to be identified. To our knowledge, our study is the first time to investigate the expression pattern of circulating miRNAs in patients with varicella. Therefore, the goals of this study were to identify a panel of serum miRNAs which are differentially expressed in varicella patients compared with non-vaccinated healthy children and to explore the potential biological functions of identified candidate miRNAs.

## 2. Results and Discussion

### 2.1. Varicella Patient Information

The basic demographic characteristics of the participants are showed in Table 1. A total of 72 participants were recruited into this study including 29 VZV infected patients (20 boys and 9 girls; median age, 0.86 ± 0.41 years) and 43 healthy children (20 boys and 23 girls; median age, 1.06 ± 0.25 years). There were no significant differences (*p* = 0.06, chi-square test) in age and sex distribution between the varicella patients and healthy children. 8 out of 29 patients (28%) were shown to be VZV-IgM positive results in serum samples by Diagnostic Kit for IgM to VZV (ELISA) (Glory Science Co., Ltd., Del Rio, TX, USA). The 29 varicella patients were diagnosed on the basis of VZV-related complications and symptoms. All these hospitalized patients had fever and skin infections. Seven patients (24%) had upper respiratory tract infection including bronchitis and cynanche. None of them had immunocompromising conditions.

**Table 1:** Demographic characteristics of varicella patients and healthy controls.

Sample Characteristic	Patients group		Healthy controls group	
	Low-Density Array study	Validation study	Low-Density Array study	Validation study
Number of participants	10	29	20	43
Gender (male/female)	5/5	20/9	10/10	20/23
Age (years, mean ± SD)	0.82 ± 0.35	0.86 ± 0.41	0.90 ± 0.27	1.06 ± 0.25
Symptoms				
Fever	10 (100%)	29 (100%)	0	0
Upper respiratory tract infection	3 (30%)	7 (24%)	0	0
Skin infection	10 (100%)	29 (100%)	0	0
VZV IgM (+)	3 (30%)	8 (28%)	0	0
Varicella vaccination	0	0	0	0

### 2.2. MiRNA Expression Profiling of VZV by TLDA in Pooled Sera

TaqMan Human miRNA Low-Density Array (TLDA) analysis was performed to identify candidate miRNAs exhibiting altered levels in response to VZV infection. Serum miRNAs of VZV infected patients were compared with those of healthy controls. Of the 667 miRNAs incorporated in the array, 274 and 304 miRNAs were detected in sera of healthy controls and patients with VZV infection, respectively. To identify VZV-specific candidate miRNAs, differential expression of miRNAs between patients and the healthy controls were required to meet two criteria: (1) CT values <35 in at least one of the two groups to enable reliable detection; and (2) miRNA levels exhibiting ≥2-fold difference between the patient and control groups [25]. A total of 247 miRNAs met these criteria, 215 of which were up-regulated and 32 were down-regulated in VZV infected patients compared with healthy controls (See Supplemental Table S1). Our studies demonstrated the potential utility of circulating miRNAs as diagnostic biomarkers of VZV infection by TLDA analysis of miRNAs that were shown to be differentially expressed in varicella patient sera. Based on the results of target gene prediction, Gene Ontology analysis showed some genes targeted by candidate miRNAs involved in immune system development, nervous system, respiratory system development and varicella development. Among these, eight miRNAs (miR-197, miR-363, miR-629, miR-132, miR-122, miR-500, miR-10a and miR-491-5p) that were significantly up-regulated (≥3.5 CT difference between the patient and control groups) were randomly selected for further analysis.

### 2.3. qRT-PCR Analysis of miRNA Expression in VZV Infected Serum

**Figure 1:**
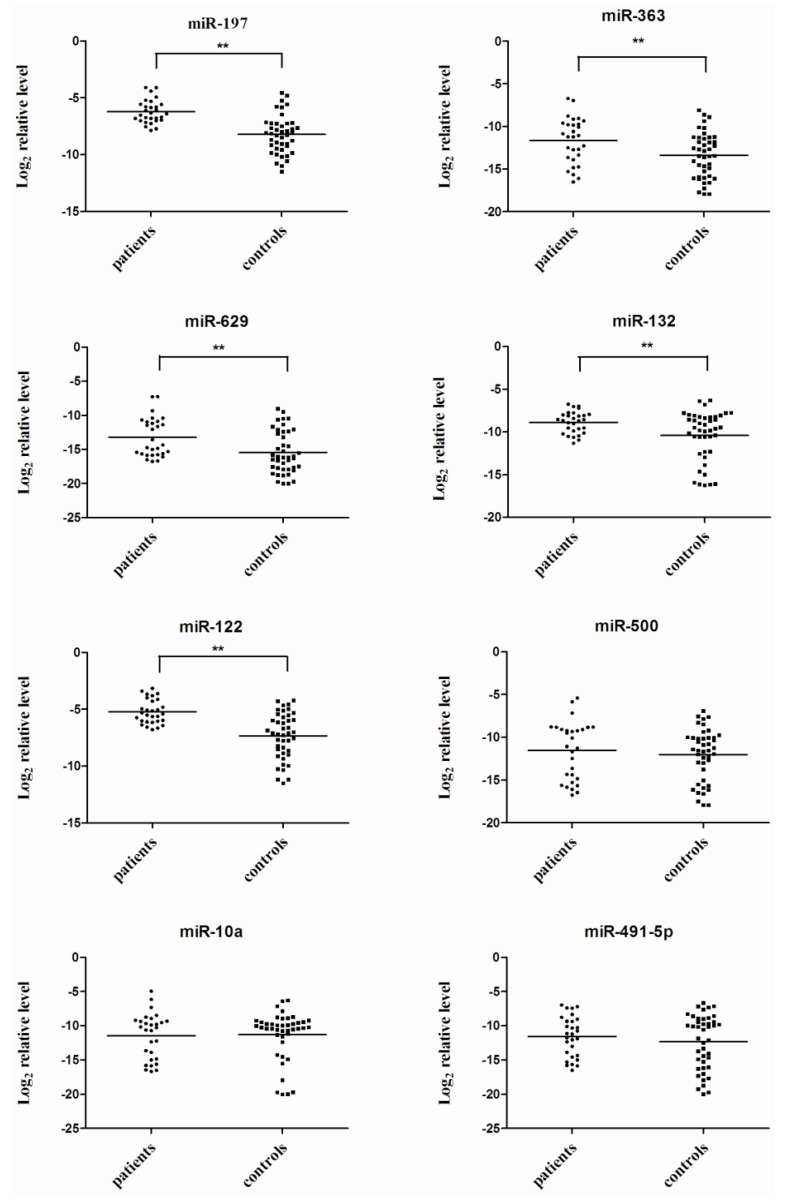
Eight serum miRNA levels in varicella patients and healthy controls were selected for verification using real-time qRT-PCR in individual varicella patients (*N* = 29) and healthy controls (*N* = 43). Serum levels of miR-197, miR-363, miR-629, miR-132 and miR-122 were significantly higher in varicella patients compared with those in the control group (**, *p* < 0.01), while no significant differences were detected in the expression of miR-500, miR-10a and miR-491-5p. Expression levels of the miRNAs are normalized to cel-miR-238 (Log_2_ relative level).

Real-time qRT-PCR (TaqMan miRNA assays) was performed in individual serum sample to further verify the expression levels of eight candidate miRNAs identified by TLDA. The eight candidate miRNAs selected for verification in serum samples from 29 patients and 43 healthy controls. The expression levels of miR-197, miR-363, miR-629, miR-132, and miR-122 showed significant up-regulation in VZV infected sera *(p* < 0.05, Student’s *t*-test), while no significant differences were detected in the expression of miR-500, miR-10a and miR-491-5p (*p* > 0.05, Student’s *t*-test) (Figure 1).

### 2.4. Evaluation of the Diagnostic Potential of miRNAs for VZV Infection

**Figure 2:**
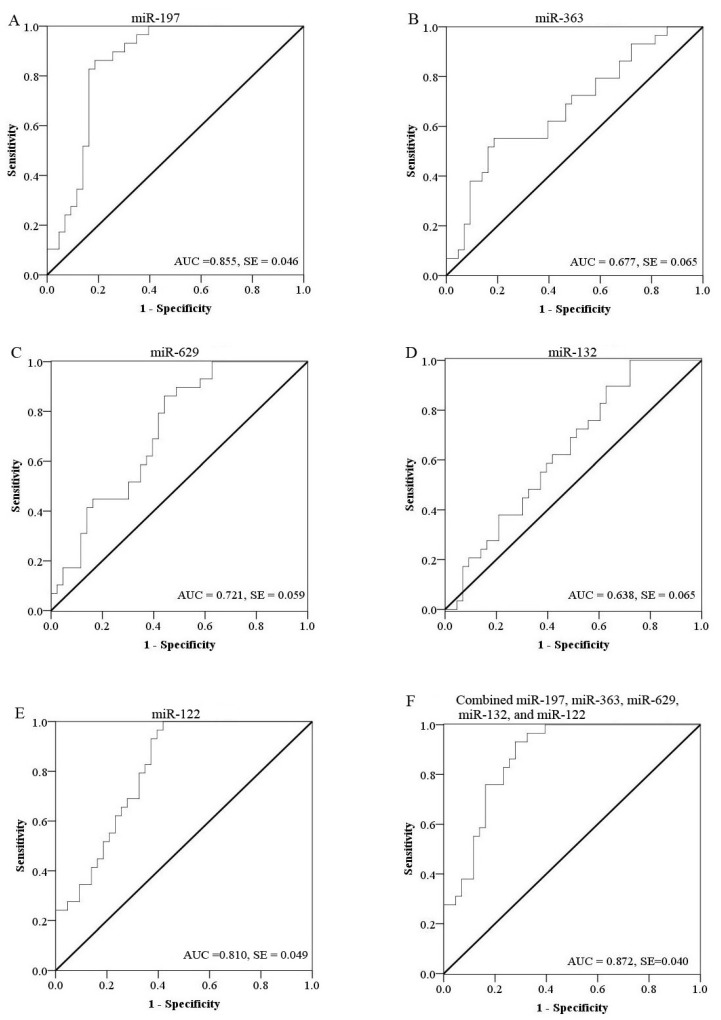
Receive operating characteristic (ROC) curves of differentially expressed miRNAs between varicella patients and healthy controls. ROC curves of miR-197 (**A**); miR-363 (**B**); miR-629 (**C**); miR-132 (**D**) and miR-122 (**E**) showed a moderate distinguishing efficiency. The combination of the five miRNAs showed a slightly higher AUC value of 0.872 (**F**).

To evaluate the efficiency of these candidate miRNAs as potential biomarkers for diagnosis of VZV infections, receiver operational characteristic (ROC) curve analysis was performed on each miRNA. The ROC curves of miR-197, miR-629 and miR-122 exhibited a moderate distinguishing efficiency with an area under curve (AUC) value of 0.855 (95% CI 0.765–0.944), 0.721 (95% CI 0.605–0.837) and 0.810 (95% CI 0.713–0.907), respectively (Figure 2A,C,E). MiR-363 (95% CI 0.550–0.804) and miR-132 (95% CI 0.511–0.764) showed significant up-regulation in the VZV infected group (*p <* 0.05), although the AUC was less than 0.7 (0.677 and 0.638, respectively) (Figure 2B,D). MiR-197 showed greater ability to distinguish VZV infection with an AUC value of 0.855. Previous studies on identifying miRNA-based biomarkers generally focused on single miRNA. For example, altered expression of miR-197 may play an important role during hepatitis B virus infection [28], as well as pancreatic cancer invasion and metastasis [29]. However, the specificity of biomarkers based on a single miRNA is very poor. In this study, we aimed to increase the diagnosis efficiency of these markers by using a combination of several host miRNAs (miR-197, miR-363, miR-629, miR-132 and miR-122). By multiple logistic regression analysis of these five differentially expressed miRNAs, the resulting ROC curve had an AUC value of 0.872 (95% CI 0.792–0.951), which reflects strong separation between the VZV infected and control samples (Figure 2F). Table 2 showed the sensitivity and specificity of each candidate miRNA and the combination of five miRNAs to diagnose varicellawith an optimal cutoff value.

At a cutoff value set at −13.03, the combined miRNAs yielded a sensitivity of 93.1% and a specificity of 72.1% (Table 2). In order to verify the specificity of the host miRNAs for VZV infection, serum pools from other microbial infections including *Bordetella pertussis* (BP), measles virus (MEV) and enterovirus (EV) were also detected by TLDA. The data of five miRNAs (miR-197, miR-363, miR-629, miR-132 and miR-122) showed significant differences between VZV and three microbial infection groups (Table 3). MiR-197 and miR-122 were generally up-regulated upon infections with bacteria or viruses. More interestingly, miR-363 and miR-629 appeared to be differentially expressed in varicella patients compared to other infectious diseases and miR-132 appeared to be similarly regulated in MV and VZV infections. Microbial infections induce changes in the host miRNA expression profile, which may also have strong effects on the development of diseases. Host miRNAs may directly or indirectly affect virus replication and pathogenesis. Since miRNA is commonly regulated in various diseases, a panel of miRNAs (miR-197, miR-363, miR-629, miR-132 and miR-122) should be a best choice to serve as potential molecular markers for VZV infection.

**Table 2:** The sensitivity and specificity of each candidate miRNA and the combination of five miRNAs to diagnose varicella with an optimal cutoff value.

miRNA	Cutoff value	Sensitivity (%)	Specificity (%)
miR-197	−7.18	86.2	81.4
miR-363	−11.23	55.2	81.4
miR-629	−15.95	86.2	55.8
miR-132	−11.80	100	27.9
miR-122	−6.82	100	58.1
Combined miRNAs	−13.03	93.1	72.1

**Table 3:** The ∆∆Ct values of three miRNAs in VZV and various microbial infections compared with controls measured by TLDA.

miRNA	VZV/control	EV/control	MEV/control	BP/control
miR-197	−10.55	−13.88	−11.87	−10.01
miR-363	−7.09	0	0	0
miR-629	−3.59	0.12	2.01	6.39
miR-132	−12.58	0	−9.73	0
miR-122	−12.77	−9.67	−16.6	−8.58

VZV: varicella-zoster virus; EV: enterovirus; MEV: measles virus; BP: *Bordetella pertussis*. The different Ct value between two groups was calculated by ∆∆Ct method: ∆Ct_patient_ = CT_target miRNA_ − CT_cel-miR-238_; ∆Ct_control_ = CT_target miRNA_ − CT_cel-miR-238_; ∆∆Ct = ∆Ct_patient_ − ∆Ct_control_.

### 2.5. Target Gene Prediction

More recently, the role of miRNA in pathogen-host interactions has attracted attention. Human miRNAs may play important roles in viral replication, limiting antiviral responses, inhibiting apoptosis and stimulating cellular growth [30]. MiRNAs are also associated with immune effects and inflammatory responses in bacterial infections [31,32]. To further investigate the possible functions of miR-197, miR-363, miR-629, miR-132 and miR-122, their predicted target genes were obtained by TargetScan algorithm (217, 196, 261, 142 and 172 respectively). Gene Ontology (GO) analysis showed some genes targeted by miR-629, miR-132 and miR-122 are involved in immune system development (6, 7 and 4 respectively). For example, BCL2 (B-cell CLL/lymphoma 2) targeted by miR-629 blocks the apoptotic death of some cells such as lymphocytes [33]. It can be speculated that this reflects the inflammatory response caused by VZV infection although the mechanism remains to be elucidated. Other genes regulated by miR-197, miR-629, miR-132, miR-363 and miR-122 are associated with nervous system, respiratory system development and lung development (Table 4).

Varicella may result in serious complications, such as pneumonia, central nervous system complications (including encephalitis), secondary bacterial infection and hemorrhagic conditions. Previous studies demonstrated that miR-197 might play an important role in the reactivation of liver inflammation by targeting IL-18, which is a key regulator in inflammation and immunity [28]. Our studies showed for the first time that higher level of miR-197 was expressed in varicella patient sera compared with healthy controls. MiR-122 has been also reported as a regulator of cell proliferation, apoptosis and migration in hepatitis B virus-related hepatocellular carcinoma [34,35,36]. Since hepatitis is a common entity of severe varicella, it can be speculated that this reflects the function of live injury caused by VZV infection. Some evidence suggested that miR-132 was an important molecule regulating embryonic stem cell differentiation into dopamine neurons [37] and miR-363 was associated with regulation of neuroblastoma tumorigenesis and metastasis [38]. It can be speculated that miR-132 and miR-363 are associated with VZV induced nervous system complications, such as encephalitis. We also found that miR-197, miR-132, miR-363 and miR-122 all target FOXP1 or FOXP2 gene, which can cooperatively regulate lung development and epithelial injury response in the lung [39,40]. Further studies are required to establish their functions in active VZV infection.

**Table 4:** The list of genes predicted to be targeted by the candidated miRNAs.

miRNAs	GO Term	Genes
miR-197	Immune system development	NA
	Nervous system	NA
	Lung development and Respiratory system development	FGF1, ADAMTS2, SPRY2, DICER1, COX1, FOXP2
miR-629	Immune system development	BCL2, CD24, CD28, SIX4, TAL1, HDAC5
	Nervous system	BCL2, SOX5, IGF1, CD24
	Lung development and Respiratory system development	ACVR2B, CUX1, HS6ST1
miR-132	Immune system development	KITLG, SMAD5, SOX4, SOX6, FOXP1, IRF1, RB1
	Nervous system	ISL1, COL4A4, MIB1, MBP, NRCAM, YWHAG
	Lung development and Respiratory system development	EP300,SMAD2, CUX1, ANO1, FOXP1, HHIP, HSD11B1, FGF7
miR-363	Immune system development	NA
	Nervous system	CDK5R1, MAP1B, ROBO1, SEMA3A
	Lung development and Respiratory system development	ATP7A, FOXP2, MAN2A1
miR-122	Immune system development	SOX6, EPO, MED1, MINK1
	Nervous system	NA
	Lung development and Respiratory system development	ADAMTS2, CUX1, DICER1, FGF1, FOXP2, SPRY2

NA: not available.

## 3. Experimental

### 3.1. Sample Collection

A total of 72 participants, including 29 patients with VZV infection and 43 healthy subjects were recruited in the Jiangsu Province between March 2009 and January 2010. The varicella-infected patients were all from the Second Hospital of Nanjing. Among them, ten varicella patients and twenty healthy controls were first recruited in the Low-Density Array study. All the participants were recruited for the quantitative RT-PCR assay in validation of the array data. Serum samples were collected from confirmed varicella patients with VZV infection (*N* = 29) who had PCR-positive results at the time of enrollment or clinical symptoms. Healthy controls were recruited at random from people undergoing a regular health check-up. Healthy controls were free of VZV infection, and showed no clinical symptoms of any infectious diseases in the routine check-up. As comparisons in array study, 60 serum specimens were collected in parallel from pediatric patients with three other microbial infections: *Bordetella pertussis* (BP), measles virus (MEV) and enterovirus (EV). Serum samples from other patients (BP, MEV and EV) were collected on the second day after admission by hospitals of Jiangsu province. All serum samples were stored at −80 °C within 4 h after collection. This project was approved by the Ethics Committee of Jiangsu Provincial Center for Diseases Prevention and Control and written informed consent was obtained from all participants.

### 3.2. RNA Extraction

Five serum pools were created (VZV group; control group; three other microbial infection groups (BP, MEV and EV) by combining 10 samples (40 µL per sample) for VZV group and 20 samples (20 µL per sample) for other groups, and mixing by inversion and 400 µL of each of these pools was used to extract RNA for assay by TaqMan Low-Density Array (TLDA). A synthetic *Caenorhabditis elegans* miRNA (cel-miR-238 (25 fmol); Takara Biotechnology Co., Dalian, China) was added into each pooled serum as an internal control before starting the isolation procedure. Isolation of total RNA from serum was carried out using mirVana PARIS kits (Ambion, Austin, TX, USA) following the instructions provided by the manufacturer with some modifications. Briefly, the pooled sera were extracted twice with an equal volume of acid-phenol chloroform and RNA was eluted with 100 µL Ambion elution solution according to the instructions provided by the manufacturer. RNA quantity and purity was measured using a NanoDrop spectrophotometer (ND-1000; ThermoScientific, Wilmington, DE, USA). RNA was extracted from individual serum sample (200 µL) used for real-time qRT-PCR assay according to a previously described method [25].

### 3.3. MiRNA Profiling Using the TaqMan Low-Density Array

MiRNA profiling assays were performed using the TLDA v2.0 (Applied Biosystems, Foster City, CA, USA). Each sample was analyzed with an A & B card for duplicate detection of a total of 667 miRNAs together with endogenous and negative controls. Briefly, 50 ng total RNA was added to 4.5 μL reverse transcription (RT) reaction mixture including 0.8 μL Megaplex RT Primer Pools A+B (10×), 0.2 μL dNTPs (100 nM), 1.5 μL MultiScribe Reverse Transcriptase (50 U/μL), 0.8 μL RT Buffer (10×), 0.9 μL MgCl_2_ (25 mM), 0.1 μL RNase inhibitor (20 U/μL) and 0.2 μL nuclease-free water. In order to increase the sensitivity of the TLDA, a pre-amplification was performed after the RT procedure using the TaqMan PreAmp Mastermix and the Megaplex PreAmp Primer Pools A+B (Applied Biosystems, Foster City, CA, USA). All reactions were carried out according to the protocols recommended by the manufacturer. RT products (2.5 µL) were pre-amplified using the Megaplex PreAmp Primers and reagents. Megaplex RT reactions were diluted 150-fold with water and 450 µL of each diluted product was combined with 450 µL TaqMan 2× Universal PCR Master Mix (No AmpErase UNG) (Applied Biosystems). The sample/master mix for each Megaplex pool (100 µL) was loaded into the array, centrifuged and mechanically sealed with the Applied Biosystems sealer device. qRT-PCR was carried out on an Applied Biosystems 7900HT thermocycler using the cycling conditions recommended by the manufacturer. Real-time PCR data were analyzed using SDS software v2.3 [41] (settings: automatic baseline; threshold, 0.2) and relative miRNA levels were calculated with the RQ Manager v1.2.1 [42] (Applied Biosystems). Serum miRNA levels were normalized to cel-miR-238 (spiked-in synthetic miRNA as an internal control). The threshold cycle (CT) values over 40 were defined as undetectable. 

### 3.4. Candidate miRNA Confirmation and Quantification by Real-Time qRT-PCR

Serum miRNA was quantified by TaqMan qRT-PCR (Applied Biosystems). Assays were performed using the RT stem-loop primer, PCR primers and probes. RT reactions were performed using the TaqMan miRNA Reverse Transcription Kit and miRNA-specific stem-loop primers in a scaled down (5 µL) RT reaction containing 1.67 µL RNA. The PCR reactions were carried out with 10 min incubation at 95 °C followed by 40 cycles of 95 °C for 15 s and 60 °C for 1 min in a final volume of 10 µL using a 7900 HT Real-Time PCR System (Applied Biosystems). A typical reaction mixture consisted of 4.5 µL diluted cDNA (1:15), 5 µL TaqMan Universal PCR Master Mix (No AmpErase UNG) and 0.5 µL TaqMan miRNA Assay primer (Applied BioSystems). Each sample was run in triplicate. The CT is defined as the fractional cycle number at which the fluorescence exceeds the defined threshold. The data were analyzed with automatic settings for assigning the baseline. The expression level of each miRNA was normalized to cel-miR-238 and was calculated using the ∆∆CT method [24].

### 3.5. Target Gene Analysis

Using TargetScan [43], the list of genes predicted to be targeted by the candidate miRNAs were obtained. The predicted target genes were analyzed for different signaling pathways or functions by DAVID server [44] with default setting [25]. 

### 3.6. Statistical Analysis

For qRT-PCR data, the relative expression level of each target miRNA (Log2 relative level) was calculated according to the difference in CT values between the target miRNA and cel-miR-238 (∆CT). Statistical analysis was performed with SPSS software version 16.0 [45]. A *p* value <0.05 was considered statistically significant. For each miRNA, a receiver operating characteristic (ROC) curve was generated. The area under curve (AUC) value and 95% confidence intervals (CI) were calculated to determine the specificity and sensitivity of VZV infection. To increase the diagnostic accuracy of combined changes in serum miRNA levels, multiple logistic regression analysis was carried out according to previously described methods [24,25].

## 4. Conclusions

In summary, TLDA assays revealed differential expression of 247 miRNAs in varicella patient sera compared to healthy controls. A combination of miR-197, miR-363, miR-629, miR-132 and miR-122 was identified as a potential non-invasive molecular marker for detection of VZV infection. The biological mechanisms of the differential expression in these miRNAs and their diagnostic potential in VZV infection require further investigation. In addition, several limitations of our study should be noted. Firstly, serum miRNA demonstrated only moderate capacity to differentiate between VZV infected patients and controls possibly because of the fact that only partial dysregulated miRNAs were evaluated in present study and other miRNA combinations might provide more efficient biomarkers. Secondly, our study represented a preliminary investigation of host responses associated with different forms of VZV infection. Biomarkers are required for different situations including protection by vaccination, other age groups among children aged 1 to 10 years, aged <20 years and adults. Therefore, additional investigations conducted in larger numbers of patients and healthy volunteers are required to validate our findings in the future.

## Supplementary Files

Supplementary File 1 Supplementary Information (PDF, 404 KB)
