# Supplementary material for: Dysregulated microRNA Expression in Serum of Non-Vaccinated Children with Varicella
Source: Viruses. 2014 Apr 22;6(4):1823–36. doi: 10.3390/v6041823 (PMC4014722; doi:10.3390/v6041823)
Supplement: Supplementary File 1 — Supplementary Information (PDF, 404 KB) [file viruses-06-01823-s001.pdf]

Supplementary Information

Table S1. Differential expressed miRNAs in VZV- infected patients compared with controls.

Detector	ΔΔCt
hsa-miR-508-5p-4395203	−16.34
hsa-miR-122-4395356	−12.77
hsa-miR-132-4373143	−12.58
hsa-miR-495-4381078	−11.55
hsa-miR-10a-4373153	−11.33
hsa-miR-425-4380926	−10.82
hsa-miR-133a-4395357	−10.67
hsa-miR-197-4373102	−10.55
hsa-miR-204-4373094	−10.54
hsa-let-7a-4373169	−10.38
hsa-miR-369-3p-4373032	−8.95
hsa-miR-487b-4378102	−8.55
hsa-miR-10b-4395329	−8.06
hsa-miR-220-4373078	−7.98
hsa-miR-342-5p-4395258	−7.78
hsa-miR-1-4395333	−7.48
hsa-miR-374b-4381045	−7.28
hsa-miR-452-4395440	−7.14
hsa-miR-363-4378090	−7.09
hsa-miR-302b-4378071	−7.06
hsa-miR-493-4395475	−6.94
hsa-let-7e-4395517	−6.94
hsa-miR-512-3p-4381034	−6.86
hsa-miR-433-4373205	−6.81
hsa-miR-500-4395539	−6.37
hsa-miR-551b-4380945	−6.23
hsa-miR-502-3p-4395194	−6.06
hsa-miR-598-4395179	−6.02
hsa-miR-376b-4373196	−5.97
hsa-miR-211-4373088	−5.92
hsa-miR-26a-4395166	−5.89
hsa-miR-885-5p-4395407	−5.85
hsa-miR-542-3p-4378101	−5.77
hsa-miR-16-4373121	−5.75
hsa-miR-518e-4395506	−5.64
hsa-miR-374a-4373028	−5.62
hsa-miR-518f-4395499	−5.53
hsa-miR-362-5p-4378092	−5.48
hsa-miR-367-4373034	−5.47
hsa-miR-519d-4395514	−5.28
hsa-miR-195-4373105	−5.28
hsa-miR-150-4373127	−5.20
hsa-miR-627-4380967	−5.18

Table S1. *Cont.*

**Detector**	**ΔΔCt**
hsa-miR-183-4395380	−5.14
hsa-miR-20a-4373286	−5.02
hsa-let-7g-4395393	−4.94
hsa-miR-486-3p-4395204	−4.92
hsa-miR-652-4395463	−4.88
hsa-miR-139-5p-4395400	−4.86
hsa-miR-223-4395406	−4.82
hsa-miR-496-4386771	−4.81
hsa-miR-365-4373194	−4.74
hsa-miR-20b-4373263	−4.73
hsa-miR-379-4373349	−4.70
hsa-miR-93-4373302	−4.62
hsa-miR-369-5p-4373195	−4.61
hsa-let-7b-4395446	−4.61
hsa-miR-26b-4395167	−4.56
hsa-miR-339-3p-4395295	−4.52
hsa-miR-501-5p-4373226	−4.49
hsa-miR-362-3p-4395228	−4.46
hsa-miR-454-4395434	−4.35
hsa-miR-15b-4373122	−4.35
hsa-miR-873-4395467	−4.29
hsa-miR-17-4395419	−4.29
hsa-miR-342-3p-4395371	−4.23
hsa-miR-126-4395339	−4.18
hsa-miR-483-5p-4395449	−4.15
hsa-miR-30b-4373290	−4.15
hsa-miR-491-5p-4381053	−4.12
hsa-miR-30c-4373060	−4.10
hsa-miR-192-4373108	−4.08
hsa-miR-886-5p-4395304	−4.06
hsa-miR-24-4373072	−4.05
hsa-miR-330-3p-4373047	−4.00
hsa-miR-186-4395396	−4.00
hsa-miR-125a-5p-4395309	−3.99
hsa-miR-340-4395369	−3.97
hsa-miR-886-3p-4395305	−3.95
hsa-miR-376c-4395233	−3.95
hsa-miR-19a-4373099	−3.93
hsa-miR-222-4395387	−3.91
hsa-miR-142-5p-4395359	−3.82
hsa-miR-106a-4395280	−3.81
hsa-miR-484-4381032	−3.79
hsa-miR-185-4395382	−3.71
hsa-miR-660-4380925	−3.68

Table S1. *Cont.*

Detector	ΔΔCt
hsa-miR-29b-4373288	−3.67
hsa-miR-28-3p-4395557	−3.65
hsa-miR-18a-4395533	−3.63
hsa-miR-146a-4373132	−3.62
hsa-miR-629-4395547	−3.59
has-miR-155-4395459	−3.58
hsa-miR-324-3p-4395272	−3.58
hsa-miR-590-5p-4395176	−3.56
hsa-miR-140-3p-4395345	−3.54
hsa-miR-628-5p-4395544	−3.53
hsa-miR-19b-4373098	−3.47
hsa-miR-28-5p-4373067	−3.44
hsa-miR-191-4395410	−3.43
hsa-miR-199a-3p-4395415	−3.43
hsa-miR-21-4373090	−3.41
hsa-miR-618-4380996	−3.40
hsa-miR-34a-4395168	−3.39
hsa-miR-489-4395469	−3.39
hsa-miR-140-5p-4373374	−3.38
hsa-miR-532-5p-4380928	−3.37
hsa-miR-146b-5p-4373178	−3.37
hsa-miR-182-4395445	−3.34
hsa-miR-298-4395301	−3.33
hsa-miR-193b-4395478	−3.32
hsa-miR-100-4373160	−3.29
hsa-miR-518a-3p-4395508	−3.29
hsa-miR-92a-4395169	−3.27
hsa-miR-655-4381015	−3.25
hsa-miR-486-5p-4378096	−3.23
hsa-miR-532-3p-4395466	−3.22
hsa-miR-451-4373360	−3.21
hsa-miR-181c-4373115	−3.16
hsa-miR-199a-5p-4373272	−3.14
hsa-miR-494-4395476	−3.12
hsa-miR-410-4378093	−3.10
hsa-miR-194-4373106	−3.10
hsa-miR-450b-3p-4395319	−3.09
hsa-miR-345-4395297	−2.96
hsa-miR-142-3p-4373136	−2.95
hsa-miR-361-5p-4373035	−2.94
hsa-miR-320-4395388	−2.94
hsa-miR-335-4373045	−2.92
hsa-miR-148b-4373129	−2.87
hsa-miR-337-5p-4395267	−2.81

Table S1. *Cont.*

Detector	ΔΔCt
hsa-miR-200c-4395411	−2.73
hsa-miR-423-5p-4395451	−2.71
hsa-miR-143-4395360	−2.69
hsa-miR-29a-4395223	−2.68
hsa-miR-106b-4373155	−2.66
hsa-miR-331-3p-4373046	−2.62
hsa-miR-455-5p-4378098	−2.62
hsa-miR-296-5p-4373066	−2.62
hsa-miR-99b-4373007	−2.57
hsa-miR-708-4395452	−2.57
hsa-miR-9-4373285	−2.55
hsa-miR-373-4378073	−2.54
hsa-miR-221-4373077	−2.54
hsa-miR-485-3p-4378095	−2.53
hsa-miR-328-4373049	−2.44
hsa-miR-130b-4373144	−2.42
hsa-miR-145-4395389	−2.42
hsa-miR-574-3p-4395460	−2.42
hsa-miR-101-4395364	−2.39
hsa-miR-517c-4373264	−2.37
hsa-miR-205-4373093	−2.30
hsa-miR-25-4373071	−2.28
hsa-miR-411-4381013	−2.28
hsa-miR-134-4373299	−2.27
hsa-miR-128-4395327	−2.24
hsa-miR-301b-4395503	−2.22
hsa-let-7d-4395394	−2.22
hsa-miR-203-4373095	−2.20
hsa-miR-503-4373228	−2.17
hsa-miR-27a-4373287	−2.16
hsa-miR-625-4395542	−2.15
hsa-miR-744-4395435	−2.13
hsa-miR-331-5p-4395344	−2.08
hsa-miR-545-4395378	−2.06
hsa-miR-579-4395509	−2.02
hsa-miR-636-4395199	−2.02
hsa-let-7c-4373167	−2.00
hsa-miR-340*-4395370	−10.71
hsa-miR-605-4386742	−9.64
hsa-miR-145*-4395260	−9.32
hsa-miR-7-1*-4381118	−9.04
hsa-miR-15a*-4395530	−8.58
hsa-miR-30d*-4395416	−7.60
hsa-miR-100*-4395253	−6.91

Table S1. *Cont.*

**Detector**	**ΔΔCt**
hsa-miR-214*-4395404	−6.79
hsa-miR-943-4395299	−6.61
hsa-miR-541*-4395311	−6.33
hsa-miR-629*-4380969	−6.25
hsa-miR-543-4395487	−6.20
hsa-miR-768-3p-4395188	−5.78
hsa-miR-411*-4395349	−5.73
hsa-miR-9*-4395342	−5.29
hsa-miR-19a*-4395535	−5.13
hsa-miR-599-4380962	−4.95
hsa-miR-624*-4380964	−4.64
hsa-miR-144*-4395259	−4.57
hsa-miR-18a*-4395534	−4.51
hsa-miR-656-4380920	−4.43
hsa-miR-26a-1*-4395554	−4.35
hsa-miR-29c*-4381131	−4.32
hsa-miR-551b*-4395457	−4.32
hsa-miR-29a*-4395558	−4.29
hsa-miR-126*-4373269	−4.21
hsa-miR-27a*-4395556	−4.05
hsa-miR-223*-4395209	−3.64
hsa-miR-425*-4395413	−3.58
hsa-miR-148b*-4395271	−3.46
hsa-miR-923-4395264	−3.38
hsa-let-7f-1*-4395528	−3.24
hsa-miR-610-4380980	−3.24
hsa-miR-190b-4395374	−3.17
hsa-miR-942-4395298	−3.13
hsa-miR-30e-4395334	−3.00
hsa-miR-99a*-4395252	−2.76
hsa-miR-30a-4373061	−2.74
hsa-miR-638-4380986	−2.72
hsa-miR-26b*-4395555	−2.69
hsa-miR-30d-4373059	−2.62
hsa-miR-30e*-4373057	−2.34
hsa-miR-30a*-4373062	−2.33
hsa-miR-93*-4395250	−2.26
hsa-miR-151-3p-4395365	−2.19
hsa-miR-632-4380977	−2.10
hsa-miR-136*-4395211	−2.00
hsa-miR-517b-4373244	2.03
hsa-miR-302c-4378072	2.23
hsa-miR-519a-4395526	2.38
hsa-miR-196b-4395326	2.50

Table S1. *Cont.*

Detector	ΔΔCt
hsa-miR-188-3p-4395217	3.31
hsa-miR-509-5p-4395346	3.33
hsa-miR-193a-3p-4395361	3.38
hsa-miR-383-4373018	3.59
hsa-miR-570-4395458	3.79
hsa-miR-516b-4395172	4.00
hsa-miR-502-5p-4373227	4.34
hsa-miR-302a-4378070	4.37
hsa-miR-449a-4373207	4.55
hsa-miR-184-4373113	4.71
hsa-miR-582-3p-4395510	5.19
hsa-miR-138-4395395	7.52
hsa-miR-382-4373019	8.16
hsa-miR-548b-5p-4395519	8.41
hsa-miR-302c*-4373277	2.76
hsa-miR-92a-1*-4395248	2.94
hsa-miR-581-4386744	2.98
hsa-miR-563-4380940	3.17
hsa-miR-374b*-4395502	3.48
hsa-miR-922-4395263	3.50
hsa-miR-34b*-4373037	3.61
hsa-miR-374a*-4395236	3.93
hsa-miR-604-4380973	4.24
hsa-miR-630-4380970	4.96
hsa-miR-589*-4380953	5.04
hsa-miR-509-3p-4395347	6.91
hsa-miR-770-5p-4395189	7.95
hsa-miR-566-4380943	11.71

The different Ct value between two groups was calculated by ΔΔCt method: $\Delta Ct_{patient} = CT_{target\ miRNA} - CT_{cel\text{-}miR\text{-}238}$; $\Delta Ct_{control} = CT_{target\ miRNA} - CT_{cel\text{-}miR\text{-}238}$; $\Delta\Delta Ct = \Delta Ct_{patient} - \Delta Ct_{control}$.

© 2014 by the authors; licensee MDPI, Basel, Switzerland. This article is an open access article distributed under the terms and conditions of the Creative Commons Attribution license (http://creativecommons.org/licenses/by/3.0/).
